# Molecular allergen sensitization profile and casein threshold determination predicting the persistence of cow's milk protein allergy in Tunisia (North Africa)

**DOI:** 10.3389/falgy.2025.1564564

**Published:** 2025-04-17

**Authors:** Yasmina Ouerdani, Imen Zamali, Yousr Galai, Ahlem Ben Hmid, Yosra Nasri, Ines Ben Sghaier, Hayet Kebaier, Hechmi Louzir, Jihene Bouguila, Nissaf Ben Alaya Bouafif, Mélika Ben Ahmed, Samar Samoud

**Affiliations:** ^1^ Laboratory of Clinical Immunology, Institut Pasteur de Tunis, Tunis, Tunisia; ^2^ Faculty of Medicine of Sousse, University of Sousse, Sousse, Tunisia; ^3^ Faculty of Medicine de Tunis, University of Tunis El Manar, Tunis, Tunisia; ^4^ Laboratory of Transmission, Control and Immunobiology of Infection, Institut Pasteur de Tunis, Tunis, Tunisia; ^5^ Faculty of Pharmacy of Monastir, University of Monastir, Monastir, Tunisia; ^6^ Department of Pediatrics, Hospital Farhat Hached, Sousse, Tunisia; ^7^ National Observatory of New and Emerging Diseases, Ministry of Health, Tunis, Tunisia

**Keywords:** cow's milk allergy, molecular allergen, specific IgE, sensitization profile, tolerance

## Abstract

**Background:**

Cow's milk protein allergy (CMPA) represents a major health concern in Tunisia, with diagnostic challenges influencing disease prognosis. Molecular allergen testing has emerged as a valuable tool to enhance diagnostic accuracy and predict disease persistence.

**Objective:**

This study aims to characterize the clinical and epidemiological features of CMPA in a Tunisian population, with a particular focus on the role of molecular allergens in assessing disease chronicity.

**Methods:**

A retrospective analysis was conducted on 262 cases of IgE-mediated CMPA diagnosed at the Pasteur Institute of Tunis between 2020 and 2023. Sensitization to molecular allergens was assessed using ImmunoCAP (Phadia 100).

**Results:**

CMPA symptoms predominantly manifested in infancy (94%, 246/262), with a male predominance (sex ratio: 1.6). Acute reactions were the most frequent presentation (69.9%, 79/113), and polysensitization was common (81%, 212/262), particularly to β-lactoglobulin. Spontaneous resolution occurred in approximately 33% of cases (29/87), with a mean age of 3 years and 8 months. Persistent CMPA was significantly associated with elevated IgE levels to whole milk, β-lactoglobulin, and casein (*p* < 0.05). ROC curve analysis identified predictive thresholds for disease persistence, including 4.2 kU/L for whole milk-specific IgE and 0.37 kU/L for casein-specific IgE (*p* = 0.006).

**Conclusion:**

Molecular allergen testing improves CMPA diagnosis and offers critical prognostic insights. The identification of IgE thresholds may facilitate early risk stratification and guide personalized management strategies.

## 1 Introduction

The prevalence of food allergies has risen significantly over the past three decades, as reported by the World Health Organization (WHO) (1). Among these, cow's milk protein allergy (CMPA) is one of the earliest to manifest, typically within the first year of life, often during the weaning period. The prevalence of CMPA in this age group is estimated between 1.6% and 3% (2). However, epidemiological data from developing countries remain scarce, with reports from Asia and North Africa primarily limited to descriptive studies and small case series.

IgE-mediated CMPA, which accounts for approximately 60% of cases, is classified as a type I hypersensitivity reaction characterized by an initial sensitization phase followed by an effector phase upon re-exposure to cow's milk proteins. Symptoms typically appear within minutes to 2 h after ingestion and can involve multiple organ systems, including the skin, gastrointestinal tract, respiratory system, and cardiovascular system (3, 4). The principal molecular allergens implicated in over 50% of cases are casein, alpha-lactalbumin, and beta-lactoglobulin (5). Accurate diagnosis is crucial for appropriate management, yet conventional methods such as clinical history, skin prick tests, and serum-specific IgE measurements present limitations in predicting the severity and persistence of CMPA. The oral food challenge remains the gold standard but is resource-intensive and carries the risk of severe reactions. Recent advancements in molecular allergology have refined diagnostic precision by enabling the measurement of specific IgE to well-characterized molecular allergens, allowing for improved risk stratification and more personalized patient management (6). However, data on sensitization patterns in different populations, particularly in North Africa, remain limited.

While CMPA generally resolves in most cases by the age of 3, with an estimated resolution rate of 87% (7), persistent forms extending beyond childhood are increasingly recognized (8, 9). Identifying predictive factors for persistent CMPA is essential for optimizing follow-up strategies and therapeutic interventions. This study provides a retrospective analysis of 262 patients diagnosed with IgE-mediated CMPA, focusing on the clinical characteristics and molecular sensitization profiles in a Tunisian population. By examining associations between sensitization patterns and disease persistence, we aim to improve early risk assessment and contribute to a more tailored diagnostic and management approach.

## 2 Methods

### 2.1 Patients and study design

**Figure 1:**
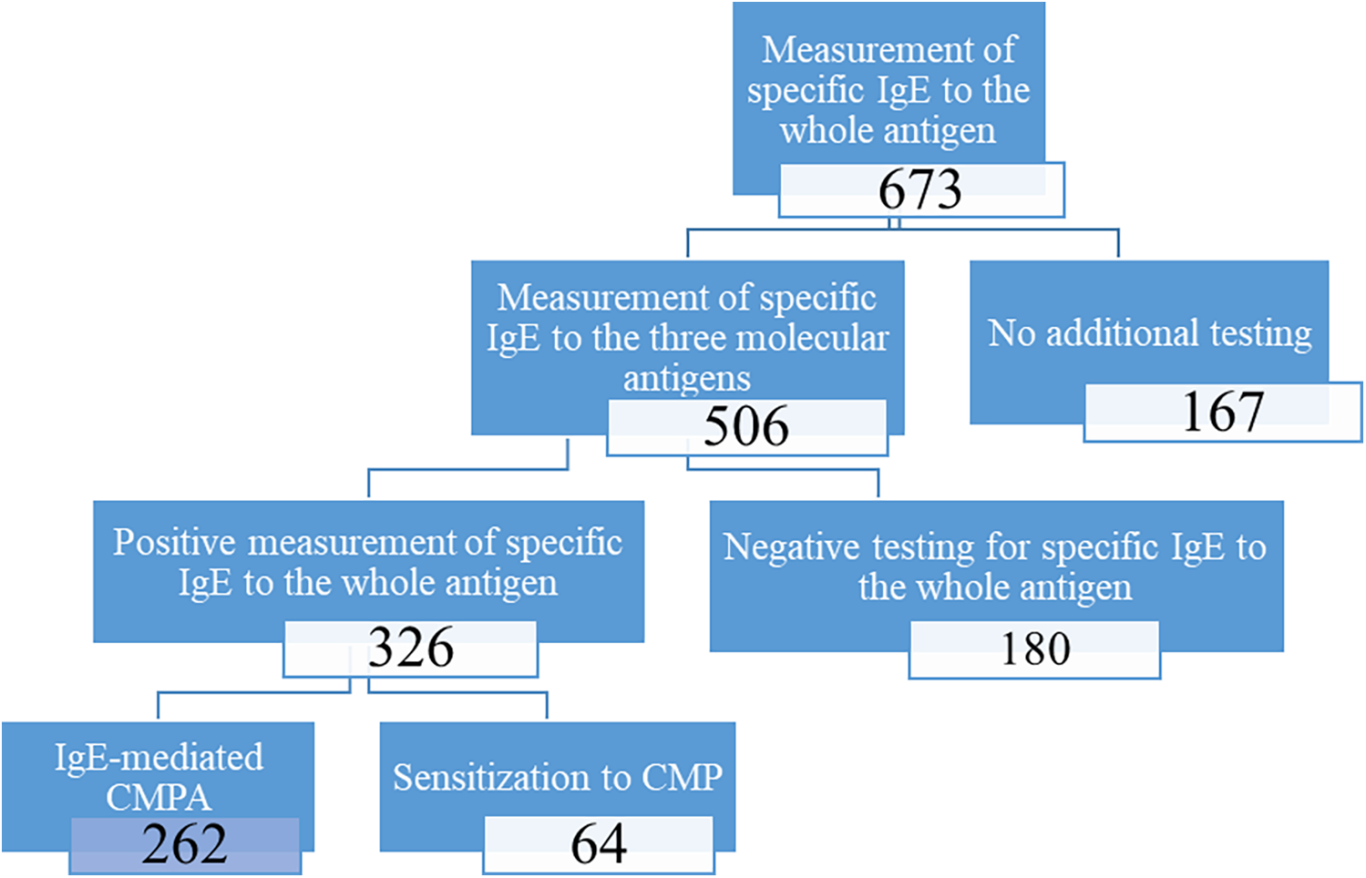
Algorithm for cow's milk protein allergy patient's inclusion.

This was a retrospective study conducted at the clinical immunology department of the Pasteur Institute of Tunis from January 2020 to August 2023. A total of 673 patients exhibiting clinical signs suggestive of CMPA, regardless of whether they had undergone other allergological tests, were referred to the institute for biological assessment. We identified all patients diagnosed with CMPA. Inclusion criteria for the study were patients with IgE levels exceeding 0.1 kU/L for the whole cow's milk antigen and at least one major recombinant molecular antigen. Patients with incomplete medical records and specimens with issues in the pre-analytical phase were excluded from the study. Non-IgE-mediated and mixed forms of CMA are not considered in this study, as our focus is exclusively on IgE-mediated allergic mechanisms. Figure 1 below shows the decisional algorithm we used to select patients according to inclusion and exclusion criteria.

Tolerance was assessed after at least 6 months on a strict elimination diet and had to meet at least one of the following criteria:

• No symptoms during a hospital-based oral food challenge.
• Specific IgE levels dropping below 0.1 kU/L.
• No reactions to accidental exposures in the past year.

In our study, persistent cow's milk protein allergy (CMPA) was defined as the reappearance of CMPA-related clinical symptoms on at least three distinct occasions, documented beyond the age of 2 years. Patient demographics, clinical characteristics, IgE specific measurements, and treatment history were extracted from electronic medical records.

### 2.2 IgE analysis

All biological analyses were conducted using ImmunoCAP tests with the Phadia® 100 system (ThermoFisher Scientific) to assess specific IgE levels for the whole cow's milk antigen (f2) and for the three key recombinant molecular allergens: Alpha-lactalbumin (f76: nBosd4), beta-lactoglobulin (f77: nBosd5) and casein (f78: nBosd8). The positivity threshold was set at ≥0.1 kU/L.

### 2.3 Statistics

Data were analyzed using SPSS 17.0 and GraphPad Prism 9.0. Percentages were used for categorical variables and compared with the Chi-square test. The Kolmogorov–Smirnov test assessed normality. Non-Gaussian data were expressed as median (25th–75th percentile) and compared with the Mann–Whitney *U* test, while Gaussian data were expressed as mean ± Standard deviation (SD) and compared with the Student's *t*-test. ROC curves assessed IgE levels' ability to distinguish persistent CMPA. The discriminatory threshold was determined using the Youden index, defined as J = sensitivity + specificity—1. The optimal threshold was selected as the value corresponding to the maximum J. The area under the curve (AUC) was calculated, and its statistical significance was tested against the null hypothesis (AUC = 0.5, indicating no discrimination) using the default nonparametric method in SPSS. Logistic regression models evaluated associations, with results reported as odds ratios (OR) and 95% confidence intervals. For the multivariable analysis, we applied forward logistic regression, including only significant variables in the final model. Significance was set at *p* < 0.05.

### 2.4 Ethics

Ethical approval for this study was obtained from the faculty of medicine of Sousse (CEFMSo_0002_2025). Patient confidentiality was maintained throughout the study, and all data were anonymized before analysis.

## 3 Results

### 3.1 Epidemiological characteristics

Our study cohort included 262 confirmed CMPA cases from 673 suspected patients. Among them, 62.2% (163/262) were male, and 37.8% (99/262) were female. Eighty percent of patients presented symptoms within the first 6 months of life, with a median onset age of 3 months. The median age at diagnosis was 1.25 years, ranging from 4 days to 35 years. Breastfeeding data were available for 80 patients, of whom 14 (17.5%) were not breastfed. Among the remaining patients, 75.7% (50/66) were exclusively breastfed for less than 6 months, with 40.9% (27/66) breastfed for less than 3 months. Delivery method data were available for 70 patients, with a majority (68.6%) born via cesarean section. Formula milk was introduced to 56 of 68 infants (82.4%) within the initial days or hours after birth. Asthma associated with CMPA was observed in 9 out of 70 patients. Family history of atopy was documented for 70 patients, among whom 48 (68.6%) reported atopy, mainly respiratory allergies such as allergic rhinitis and/or asthma. Additionally, 19 (27.1%) reported a family history of CMPA.

### 3.2 Clinical manifestations

Symptoms were documented for 113 patients (Table 1). Cutaneous manifestations were the most common, with rashes observed in 48.6% (55/113) and angioedema in 38.9% (44/113). Digestive symptoms were reported in 23% of cases, while respiratory symptoms accounted for 3.5%. Hospitalization was required in 20.9% of cases. Acute symptoms appeared within minutes to 2 h in 69.9% (79/113) of cases, while delayed symptoms occurred in 30.1% (34/113). Single organ involvement was noted in 47.5% (54/113), whereas 52.5% (59/113) exhibited multi-organ involvement.

**Table 1:** Clinical characteristics of patients with cow's milk protein allergy.

Symptoms	Patients *N* = 113 n (%)
Angioedema	44 (38.9)
Cutaneous	73 (64.6)
Skin rash	55 (75.3)
Eczema	8 (11.0)
Urticaria	10 (13.7)
Respiratory	4 (3.5)
Gastrointestinal	26 (23)
Diarrhea	4 (15.4)
Vomiting	15 (57.6)
Diarrhea and vomiting	6 (23)
Bloody stools	1 (3.8)
Anaphylactic shock	3 (2.6)

Asthma (*p* = 0.093), additional food allergies (*p* = 0.23), sex (*p* = 0.47), family history of atopy (*p* = 0.21), breastfeeding (*p* = 0.38), and formula feeding history (*p* = 0.18) were not predictive of oral tolerance acquisition. Due to the retrospective nature of the data collection, there were missing data for several variables included in the analysis. As a result, the denominator varies for each factor, and the number of patients analyzed for each variable is lower than the total cohort of 262.

### 3.3 Non-biological tests in allergology

Among 67 patients, 43 (64.2%) had a skin prick test, while 14 (20.9%) underwent an oral food challenge (OFC).

### 3.4 IgE-sensitization at diagnosis

**Figure 2:**
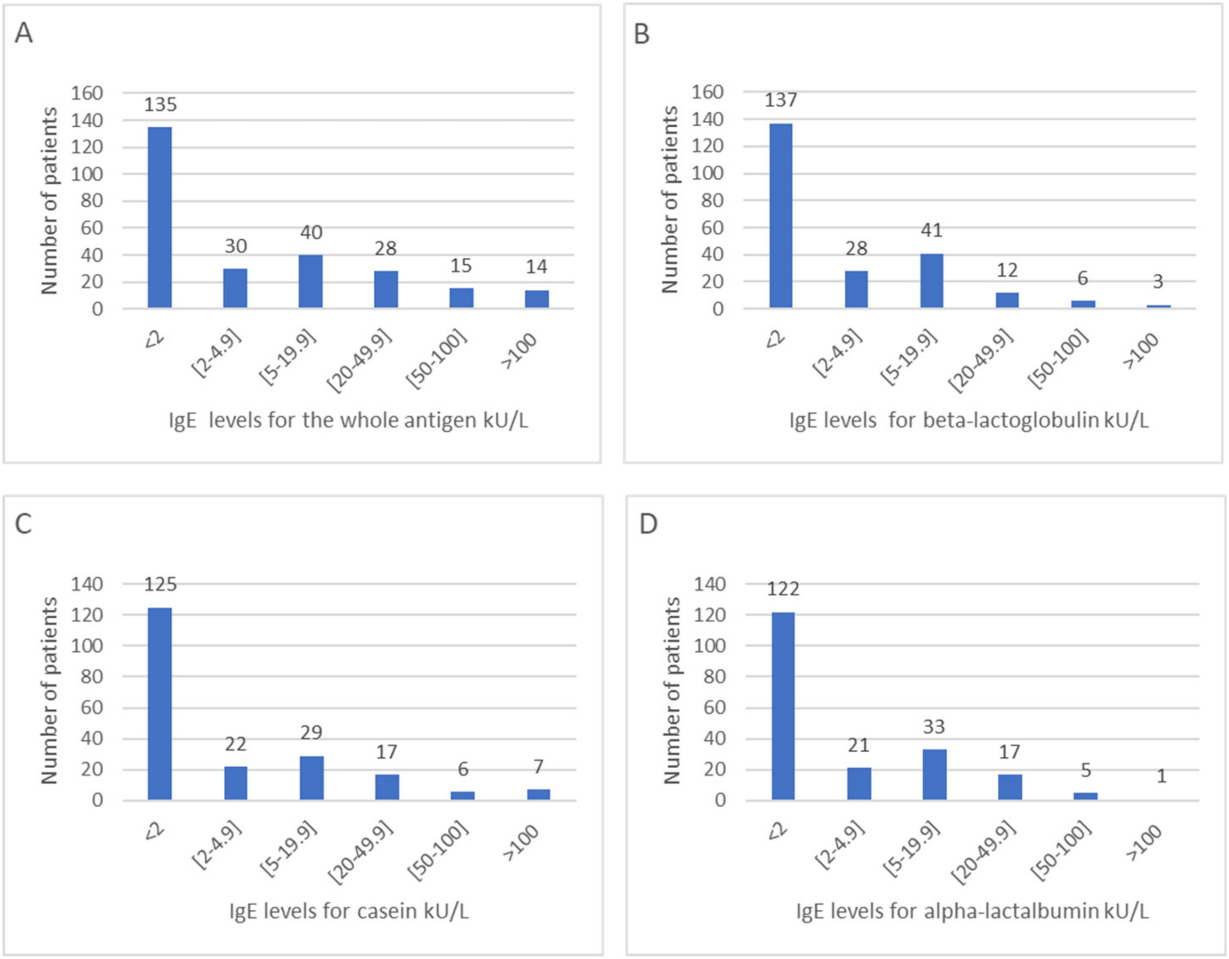
Distribution of IgE levels to cow's milk and its components in cow's milk protein allergy patients: **(A)** whole antigen (*N* = 262), **(B)** beta-lactoglobulin (*N* = 227), **(C)** casein (*N* = 206), **(D)** alpha-lactalbumin (*N* = 199).

Serum specific IgE to cow's milk protein data were available for all 262 patients (Table 2). A majority (81%) were sensitized to multiple proteins. Beta-lactoglobulin demonstrated the highest rate of sensitization, affecting 86% of patients. IgE levels for the whole antigen, alpha-lactalbumin, beta-lactoglobulin, and casein are shown in Figure 2. For each allergen, we included all patients with a positive result (>0.1 kU/L), which explains the differences in subgroup sizes: whole antigen (*N* = 262 patients), β-lactoglobulin (*N* = 227 patients), casein (*N* = 206 patients), and α-lactalbumin (*N* = 199 patients).The mean values of IgE for the whole antigen, alpha-lactalbumin, beta-lactoglobulin, and casein were 15 kU/L (0.37–16), 5.59 kU/L (0.11–2.85), 6.02 kU/L (0.14–4.33), and 7.99 kU/L (0.11–3.95), respectively.

**Table 2:** Sensitization profile to recombinant molecular allergens in cow's milk protein allergy patients.

Sensitization to recombinant molecular antigens	Patients *N* = 262 *n* (%)
A single molecular antigen	50 (19.1)
Alpha-lactalbumin	7 (2.7)
Beta-lactoglobulin	25 (9.5)
Casein	18 (6.9)
Two molecular antigens	54 (20.6)
Alpha-lactalbumin and beta-lactoglobulin	24 (9.2)
Casein and alpha-lactalbumin	10 (3.8)
Casein and beta-lactoglobulin	20 (7.6)
Three molecular antigens	158 (60.3)

In 26 out of 30 patients, additional food or respiratory allergies were diagnosed based on clinical findings and physician requests, with egg allergy being the most common (19/26, 73%) (Table 3).

**Table 3:** Prevalence of additional food or respiratory allergies in cow's milk protein allergy patients.

Additional allergies	Patients *N* = 26 *n* (%)
Peanuts	1 (4)
Wheat	2 (7)
Beef	4 (15.4)
Fish	4 (15.4)
Egg	19 (73)
Egg white	17 (89.5)
Egg yolk	1 (5.2)
Egg white and egg yolk	1 (5.2)
Cat and dog hair	2 (7)
Mites	4 (15.4)
Pollen	3 (11.5)

### 3.5 Treatment

Treatment strategies were documented for 80 patients. All patients adhered to a strict elimination diet. Among them, 66 patients (82.5%) continued breastfeeding, while 33 (41.25%) were switched to extensively hydrolyzed formulas (eHF). Additionally, two patients received rice milk, two were given soy milk, and one was prescribed a synthetic amino acid formula.

### 3.6 Cow's milk protein allergy resolution

Clinical tolerance data were available for 87 patients. Only 29 patients (33.3%) achieved milk tolerance, with a median age of 3 years and 8 months (range: 7 months to 10 years). Tolerance was confirmed through oral food challenges (6 patients), specific IgE levels dropping below 0.1 kU/L (13 patients), and the absence of reactions to accidental exposures (10 patients).

### 3.7 Predictive markers of persistence

**Figure 3:**
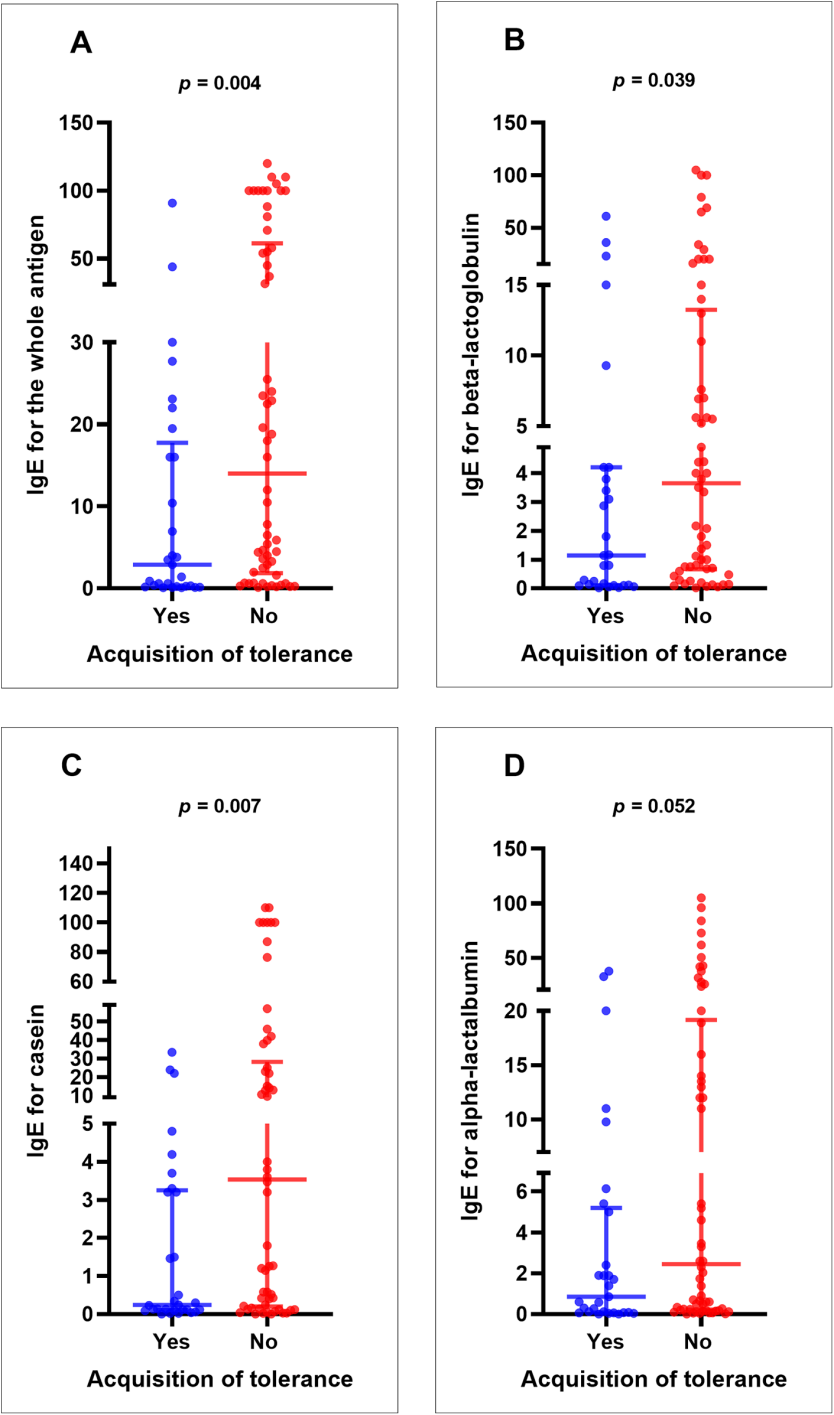
IgE levels to cow's milk and its components based on tolerance acquisition (Yes) or persistent sensitization (no) (*N* = 87): **(A)** whole antigen, **(B)** beta-lactoglobulin, **(C)** casein, **(D)** alpha-lactalbumin. *p*- values were calculated using the nonparametric Mann–Whitney *U* test.

Patients with persistent CMPA exhibited significantly higher cow's milk-specific IgE levels at diagnosis compared to those who developed tolerance (median: 14 kU/L vs. 2.9 kU/L; *p* = 0.004). This pattern was also observed for beta-lactoglobulin (median: 3.6 kU/L *vs*. 1.14 kU/L; *p* = 0.039) and casein-specific IgE levels (median: 3.5 kU/L *vs*. 0.24 kU/L; *p* = 0.007), but not for alpha-lactalbumin (median: 2.4 kU/L vs. 0.85 kU/L; *p* = 0.052) (Figure 3).

**Figure 4:**
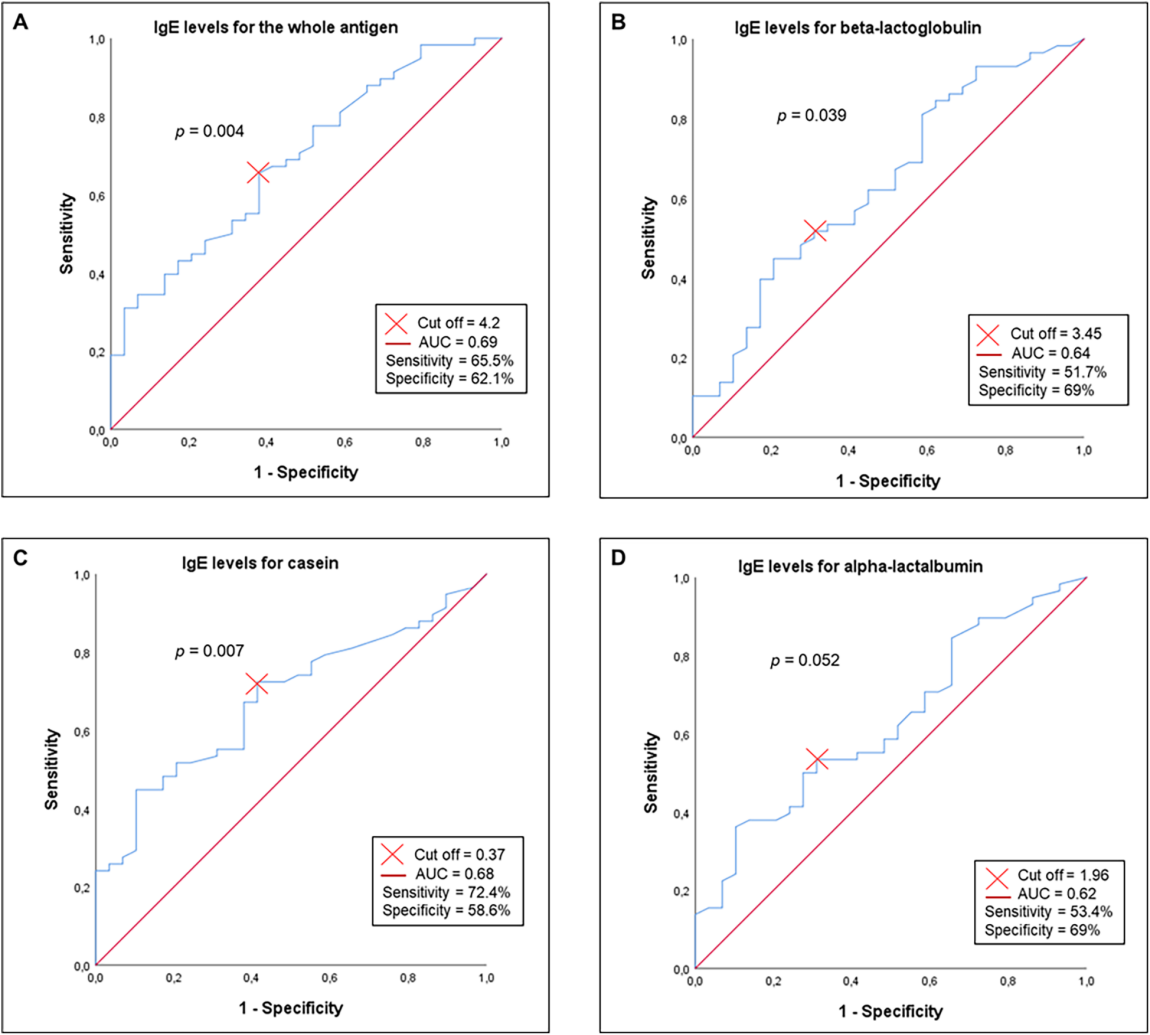
Receiver operating characteristic (ROC) curves illustrating the ability of IgE levels to discriminate persistent cow's milk protein allergy from cow's milk and its components (*N* = 87): **(A)** whole antigen, **(B)** beta-lactoglobulin, **(C)** casein, **(D)** alpha-lactalbumin. The discriminatory threshold was determined using the Youden index. *p*- values were generated using SPSS's default nonparametric method for ROC analysis.

ROC curve analysis demonstrated that specific IgE levels for the whole antigen, beta-lactoglobulin, and casein reliably distinguished between persistent CMPA and cases achieving tolerance (*p* < 0.05). However, alpha-lactalbumin-specific IgE levels lacked (*p* > 0.05) discriminatory power (Figure 4).

Univariate logistic regression identified higher specific IgE levels to whole antigen (*p* = 0.016) and casein (*p* = 0.006) as significant predictors of persistent CMPA, with levels surpassing ROC curve thresholds (OR >1, *p* < 0.05). No significant associations were observed for alpha-lactalbumin or beta-lactoglobulin (Table 4).

**Table 4:** Predictors for persistent cow's milk protein allergy by univariate logistic regression analysis.

Cow's milk protein	Threshold	OR >Threshold vs. <Threshold (95% CI)	*p*
IgE for whole antigen	4.20	3.10 (1.23–7.84)	0.016*
Alpha-lactalbumin	1.96	2.55 (0.99–6.54)	0.051
Beta-lactoglobulin	3.45	2.38 (0.93–6.10)	0.071
Casein	0.37	3.72 (1.46–9.50)	0.006*

*Statistically significant (*p* < 0.05).

For multivariable analysis, we performed forward logistic regression by including one by one the following explicative variables: age at diagnosis, gender and IgE levels exceeding ROC threshold for the whole antigen, beta-lactoglobulin, alpha-lactalbumin and casein, to assess their association with persistence (dependent variable).

After adjustment on gender and age at diagnosis, only casein was significantly associated with persistence and was considered in the final model (Table 5)**.**

**Table 5:** Predictors for persistent cow's milk protein allergy by multivariate forward logistic regression analysis.

Parameters	OR (95% CI)	*p*
Gender	2.36 (0.79–7.04)	0.121
Age at diagnosis	1.08 (0.88–1.31)	0.437
Casein exceeding ROC threshold	3.60 (1.31–9.92)	0.013*

*Statistically significant (*p* < 0.05).

## 4 Discussion

**Figure 5:**
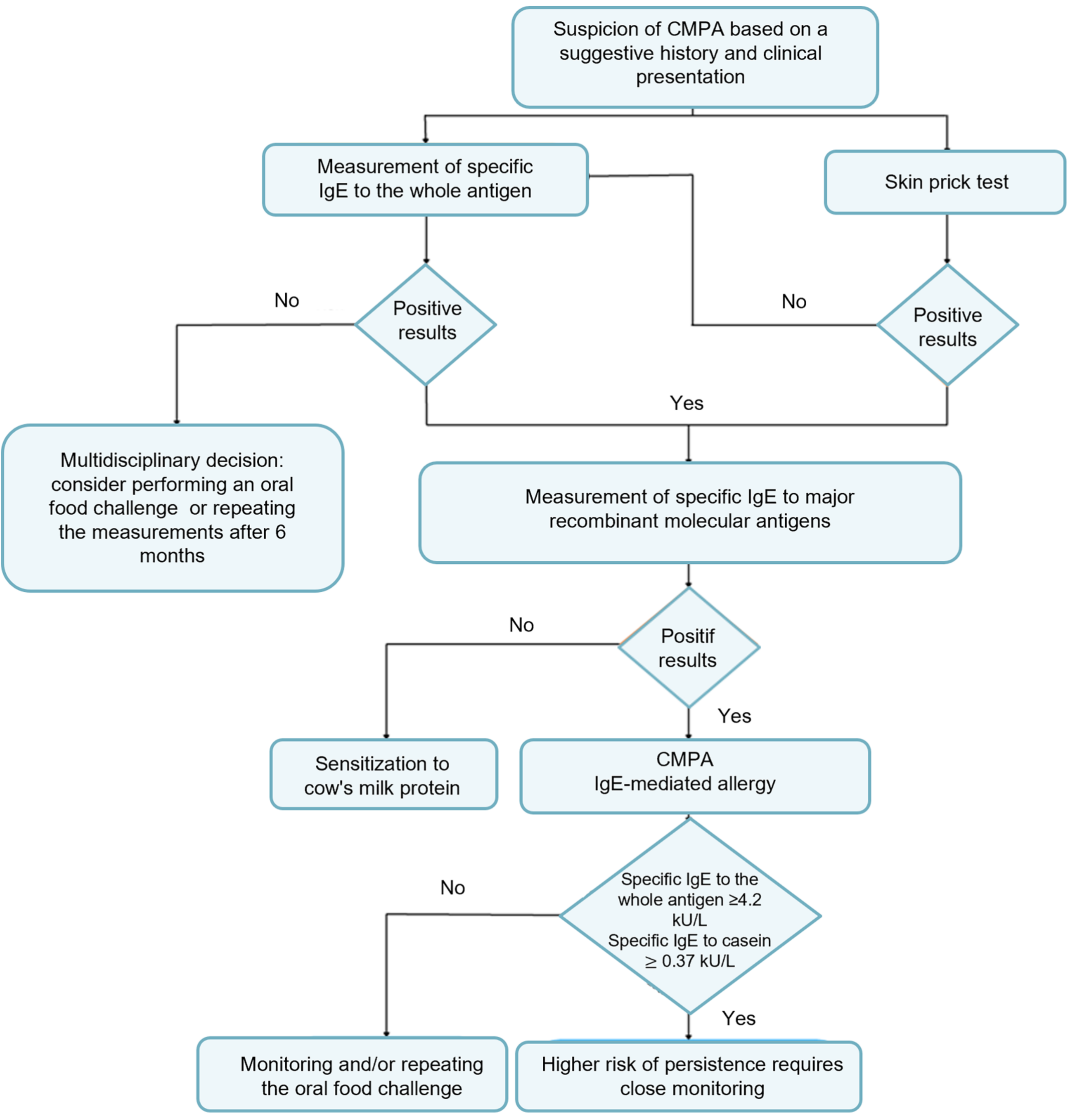
Personalized decision algorithm for managing and monitoring cow's milk protein allergy.

Cow's milk protein allergy (CMPA) remains a significant public health concern in Tunisia, with a rising prevalence yet limited available data on its clinical characteristics and management (10, 11). Our retrospective study of 262 patients with IgE-mediated CMPA provides valuable insights into the disease's clinical presentation, diagnostic challenges, treatment strategies, and patient outcomes within a real-life Tunisian setting. A key finding in our cohort was the higher prevalence of CMPA in males, consistent with previous reports (12). The median age at symptom onset aligned with the typical childhood presentation (13, 14), with symptoms frequently appearing upon the first introduction of cow's milk during weaning, usually within the first year of life. Our analysis underscores a diagnostic delay in nearly one-third of patients, with diagnoses occurring after the age of 2. This delay was attributed to subtle initial symptoms, frequent misdiagnoses, postponed medical consultations, and a lack of systematic allergological testing. These findings highlight the urgent need for greater awareness and earlier recognition of CMPA to ensure timely and effective management. As recommended by the WHO and UNICEF, initiating breastfeeding within the first hour of birth and maintaining exclusive breastfeeding for the first 6 months are crucial (15). Breast milk, rich in hormones, cytokines, and immune cells, plays a protective role in reducing allergy risks, particularly in high-risk infants (16). However, Tunisia records one of the lowest breastfeeding rates globally, with over 60% of newborns not breastfed within the first hour and 80% not exclusively breastfed for the first 6 months (17). Furthermore, the high rate of cesarean deliveries in our series may partly explain the observed early cow's milk sensitization, which can occur within the first hours or days of life. A strong familial atopy component was evident in our cohort, with CMPA being particularly prevalent in children with a family history of allergies. This finding aligns with the study by Saarinen et al., which reported that the risk of CMPA doubles when a parent or sibling has allergies (8). As expected, we observed an association between CMPA and asthma, with prevalence rates ranging from 30% to 50% in various studies (18, 19). In a cohort of 100 patients, Bishop et al. reported that 40% of individuals with CMPA developed asthma within 5 years (20). This association may contribute to the severity of clinical symptoms and the persistence of allergic manifestations (18, 21). In our study, while asthma appeared underdiagnosed, the lack of long-term follow-up prevents definitive conclusions. The literature consistently identifies skin symptoms as the most frequent clinical presentation of CMPA (70%–75%), followed by digestive (34%) and respiratory (8%) manifestations. More than 50% of cases present with skin erythema, with or without angioedema, while 10%–15% exhibit only localized signs, such as perioral erythema (9, 12, 19, 22). Early signs may also include bottle refusal and irritability. Severe manifestations requiring hospitalization were observed in 20.9% of cases, including instances of anaphylactic shock, underscoring the critical need for early diagnosis and intervention. Notably, food-related anaphylaxis, with cow's milk being a primary trigger, is three times more common in infants under 4 years old (23). Diagnostic methods for CMPA have evolved significantly. While the oral food challenge (OFC) was historically considered the gold standard, its limitations in predicting the severity of future reactions and the risk of severe secondary responses make it less suitable as a first-line diagnostic tool. Instead, a suggestive clinical presentation combined with a positive skin prick test and/or elevated specific IgE levels is now deemed sufficient to confirm the diagnosis and initiate an elimination diet (24). The OFC has limited predictive value regarding reaction severity, with only 4.4% of the variance in clinical response intensity attributed to the reactive dose (25). The skin prick test remains a highly sensitive tool for diagnosing immediate hypersensitivity, but its specificity is lower, with a positive predictive value of only 56% (26, 27). Our findings highlight variability in diagnostic practices, underscoring the need for a national consensus on CMPA management. The advent of molecular diagnostic tools such as ImmunoCAP has transformed the diagnostic landscape. ImmunoCAP provides a highly specific method for quantifying specific IgE levels, offering valuable insights into sensitization markers and aiding in food allergy confirmation (28). Renowned for its high specificity and a positive predictive value exceeding 90%, quantitative ImmunoCAP analysis has become a reference technique in food allergy diagnostics (29). In our cohort, beta-lactoglobulin was implicated in 86% of cases; however, casein has emerged as the dominant allergen due to its physicochemical stability, which allows it to retain IgE-binding capacity even after extensive heating (30, 31). This stability may explain the severity and persistence of CMPA in certain patients, particularly those reacting to both raw and cooked milk. The resilience of casein could contribute to both immediate and delayed severe reactions, potentially influencing the chronicity of CMPA. Host's study reported that 18% of children with CMPA developed additional allergies (32). In our analysis, we identified 26 confirmed cases of allergies beyond CMPA, with 65.4% linked to egg white. Similarly, an Alergólogica study highlighted that milk and eggs are the most common allergens in children under 5 years old (33). The management of CMPA in our cohort adhered to international guidelines, emphasizing a strict elimination diet and encouraging breastfeeding whenever possible (34). Dietary counseling focused on identifying hidden sources of CMP, while substitute formulas were provided for infants under 2 years of age or those experiencing growth delays (35). Cow's milk hydrolysates were the most frequently used alternative and proved effective in most cases. However, 11.3% of patients showed poor tolerance due to residual proteins. These hydrolysates contain 95% of peptides below 1,500 Da and only 0.5% exceeding 6,000 Da, with higher molecular weights being associated with increased antigenic potential and reduced safety (36). Alternatives included amino acid-based formulas, rice milk, and soy milk (37). Oral immunotherapy (OIT) has shown promise for managing persistent CMPA in children older than 5 years by gradually increasing milk tolerance. After 1 year of treatment, OIT led to successful outcomes in 36% of cases (38, 39). However, its efficacy decreased in patients with elevated IgE levels and carried a risk of severe side effects (40). A decline in IgE levels over time is often predictive of tolerance development, whereas increasing levels indicate sustained allergen reactivity (41). In our cohort of 87 children, allergy resolution occurred in one-third of cases at an average age of 3 years and 8 months (44 months). These findings contrast significantly with a widely referenced study reporting that 75% of children with IgE-mediated CMPA achieved tolerance by age 3 (7). Similarly, a large study by Høst et al. involving 1,749 Danish children reported tolerance rates of approximately 45%–50% at 1 year, 60%–75% at 2 years, and 85%–90% at 3 years (32). However, our results align more closely with studies reporting lower tolerance rates. A Tunisian study of 37 patients with an average follow-up of 40.2 months found tolerance rates of 13.7% at 1 year, 60.8% at 2 years, 74.6% at 3 years, and 78.8% at 4 years (10). Similarly, a 2007 study by Skripack et al. reported that only 19% of 807 allergic children outgrew CMPA by age 4, with rates increasing to 42% by age 8, 64% by age 12, and 79% by age 16 (9). In another follow-up study of 79 children with CMPA, 17.7% achieved tolerance by age 4, 31.6% by age 6, 39.2% by age 8, and 44% still had CMPA at age ten (19). In our cohort, one patient developed tolerance at age 10, underscoring that resolution can extend into adolescence. However, our findings must be interpreted cautiously, as the retrospective study design and loss of follow-up data limit the ability to draw definitive conclusions. Specific IgE levels emerged as the most reliable predictors of CMPA resolution in our study. Patients with persistent CMPA exhibited significantly higher IgE levels to whole antigen, beta-lactoglobulin, and casein compared to those who developed tolerance (*p* < 0.05). These findings are consistent with prior studies linking elevated IgE levels to a poorer prognosis (9, 41, 42). Payot et al. reported that tolerant children had significantly lower IgE levels for whole milk (2 kU/L vs. 11.5 kU/L, *p* < 0.0001) and casein (1 kU/L vs. 16 kU/L, *p* = 0.0014) (42). Thus, quantitative IgE analyses, rather than semi-quantitative methods, are essential for accurate monitoring and personalized treatment planning. Through ROC curve analysis, our study identified a predictive threshold for persistent CMPA at 4.2 kU/L for whole antigen-specific IgE. A 2012 German study reported similar findings, showing that children with IgE levels below 5 kU/L achieved tolerance within 18 months, whereas higher levels prolonged this period to 33 months (43). In addition, we identified a predictive threshold for casein-specific IgE at 0.37 kU/L. A retrospective analysis of 72 patients undergoing OFC highlighted casein-specific IgE as the most robust predictor of persistence, with an AUC of 0.976. At a threshold of 0.95 kU/L, sensitivity and specificity were 88.9% and 90.9%, respectively (44). Other studies have also confirmed the predictive value of casein-specific IgE, though reported thresholds vary (45, 46). Interestingly, the lower casein-specific IgE thresholds observed in our study may be unique to Tunisian populations, underscoring the need for region-specific diagnostic approaches and personalized management strategies. To further validate the predictive value of specific IgE levels, we performed a multivariable analysis using forward logistic regression. By sequentially incorporating age at diagnosis, gender, and IgE levels exceeding the ROC-defined thresholds for whole antigen, beta-lactoglobulin, alpha-lactalbumin, and casein, we assessed their association with CMPA persistence. After adjusting for gender and age at diagnosis, only casein-specific IgE remained significantly associated with persistence and was retained in the final model. These results reinforce the central role of casein sensitization in CMPA prognosis, highlighting the need for its systematic evaluation in clinical practice. The integration of molecular diagnostic tools, particularly quantitative casein-specific IgE measurement, could enhance risk stratification and guide personalized management strategies. Our findings provide further support for developing a structured decision-making framework to optimize CMPA diagnosis and follow-up. To assist clinicians in managing CMPA, we propose an updated decision tree model (Figure 5) that incorporates key diagnostic steps, biomarkers, and clinical symptoms. This model provides a systematic, evidence-based approach to diagnosis and treatments, aiming to reduce diagnostic delays and improve patient outcomes.

While molecular diagnostics have significantly advanced, growing evidence suggests that the gut microbiome plays a crucial role in the development and persistence of food allergies, including CMPA. A recent study (47) highlights the microbiome's impact on immune modulation and suggests that incorporating microbiome data into diagnostic models could enhance patient stratification and treatment personalization. Although our study did not directly investigate the microbiome, these findings offer valuable insights for future research. Integrating microbiome-based biomarkers alongside IgE measurements could refine diagnostic models and facilitate tailored treatment strategies, aligning with the shift toward personalized medicine (47).

Finally, our study's retrospective design and incomplete follow-up data represent notable limitations. While these constraints are inherent to real-world data, they also highlight gaps in CMPA management in Tunisia. By exposing these shortcomings, we aim to raise awareness among clinicians about the importance of more rigorous data collection and better standardization of practices. To address these gaps, a prospective study is currently underway to validate our findings and further explore the interplay between the microbiome and personalized treatment strategies in CMPA management.

## 5 Conclusions

Our retrospective analysis offers valuable insights into the clinical characteristics, diagnostic methods, therapeutic approaches, and outcomes of CMPA in a Tunisian population, representing a North African context. Notably, we identified a predictive threshold for persistent CMPA based on specific IgE levels, with an exceptionally low threshold for casein-specific IgE. These findings underscore the critical importance of personalized diagnostic protocols and a multidisciplinary approach to enhance the management of this complex allergy. Further research is essential to advance our understanding of CMPA and refine diagnostic tools and treatment strategies, ultimately improving patient care and outcomes.

## Data Availability

The original contributions presented in the study are included in the article/Supplementary Material, further inquiries can be directed to the corresponding author.
